# Trends in fracture development of the upper extremity in Germany—a population-based description of the past 15 years

**DOI:** 10.1186/s13018-020-1580-4

**Published:** 2020-02-21

**Authors:** P. Hemmann, P. Ziegler, C. Konrads, A. Ellmerer, T. Klopfer, A. J. Schreiner, C. Bahrs

**Affiliations:** Department of Traumatology and Reconstructive Surgery, BG Trauma Center Tuebingen, Eberhard Karls University Tuebingen, Schnarrenbergstrasse 95, 72076 Tuebingen, Germany

## Abstract

**Background:**

Recent studies investigating fracture development in Germany are not available especially with regard to demographic change. The primary aim of this study was to report trends in fracture development of the upper extremity in Germany between 2002 and 2017 and to evaluate changes over time.

**Methods:**

Evaluating inpatient data from the German National Hospital Discharge Registry (International Classification of Diseases, ICD-10) between 2002 and 2017. Total count, incidences and percentage changes of the following fracture localizations were analysed: proximal humerus, distal humerus, proximal ulna, proximal radius, ulna diaphysis (including Monteggia lesion) and distal radius. Ten age groups for men and women were formed: 35–44, 45–54, 55–64, 65–74; 75–84; 85–90, and > 90 (years).

**Results:**

The total count of proximal humeral fractures increased from 40,839 (2002, men/women 9967/30,872) to 59,545 (2017, men/women 14,484/45,061). Distal humeral fractures increased from 5912 (2002, men/women 1559/4353) to 6493 (2017, men/women1840/4653). The total count of forearm fractures increased from 68,636 (2002, men/women 17,186/51,450) to 89,040 (2017, men/women 20,185/68,855). Women were affected in 70–75% of all cases with rising incidences among nearly every age group in female patients.

**Conclusion:**

Total count of nearly every evaluated fracture increased. Also, incidences increased especially in the older female age groups. Fracture development already seems to reflect demographic changes in Germany.

## Introduction

Demographic change and increased life expectancies lead to an overaged population in Germany. According to the German Federal Office of Statistics, more than 50% of the population will be older than 50 years by the year 2050 [1]. Hence, the amount of geriatric fractures will increase [2], associated with high effort and high costs for the public health care system [3]. In light of this, surgeons will be confronted with higher numbers of (low-energy-associated fractures due to age-related diseases like osteoporosis and accidental falls [4, 5]. Especially, osteoporosis, with high prevalence in people over 50 years [6], causes typical fractures of the elderly affecting the proximal humerus, distal radius, proximal femur, ankle joint, spine and pelvis [7–10]. Treatment of these fractures and comorbidities respectively represents a challenge for the attending physician.

Although, there are several studies dealing with epidemiology of hip fractures [3, 11, 12], there is no study which describes the development of fractures in Germany, especially fracture development of the upper extremity. A few epidemiological studies of other countries have been published in the last years about fracture development of the proximal and distal humerus [2, 7, 13, 14].

The goal of the present study was to analyse the epidemiological development of fractures between 2002 and 2017 in Germany. Trends in fractures of the upper extremity were evaluated for people > 35 years of age with focus on the elderly respectively by analysing the national hospital discharge diagnosis register. Moreover, the study presents the shift of prevalences and incidences in different fractures of the upper extremity.

## Patients and methods

The authors analysed nationwide data of the national hospital discharge diagnosis register between 2002 and 2017. This database is maintained by the RKI (Robert Koch Institute) and the German Federal Office of Statistics. Therefore, it includes patient data of more than 99% of all German hospitals and can provide reliable epidemiological information. Previous studies have already demonstrated the validity of this data base [15].

The International Classification of Diseases (ICD) system was used to identify relevant diagnosis. ICD-10-GM (German modification) was evaluated for the following fractures: proximal humerus/S42.2, distal humerus/S42.4, proximal ulna/S52.0, proximal radius/S52.1, ulna diaphysis/S52.2 (including Monteggia-Lesions) and distal radius/S52.5. ICD-10-GM codes have been used since 2000. To minimise documentation errors after changing to ICD-10-GM, the authors determined 2002 as starting point for further study analysis [16].

Men and women were split into age groups with a range of 10 years per age group: 35–44, 45–54, 55–64, 65–74, 75–84, 85–90, and > 90. All patients > 90 years were analysed in one group.

Furthermore, the national hospital discharge diagnosis register provides demographic data about suitable age groups for men and women for the years 2002 and 2017 and the population for both sexes for each year was used to calculate the fracture incidence per each corresponding age group (*n*/100,000/year).

We analysed the total count and incidence of several fractures of the upper extremity from the year 2002 and 2017 and evaluated differences in sex and age groups and percentage changes were evaluated 

Statistical analysis was performed with Microsoft© Office Excel 365 ProPlus (Microsoft Corporation, Redmond, USA). Descriptive statistical analysis displays prevalences and incidences (*n*/100,000/year) as well as diagrams.

## Results

### Proximal humerus

**Fig. 1:**
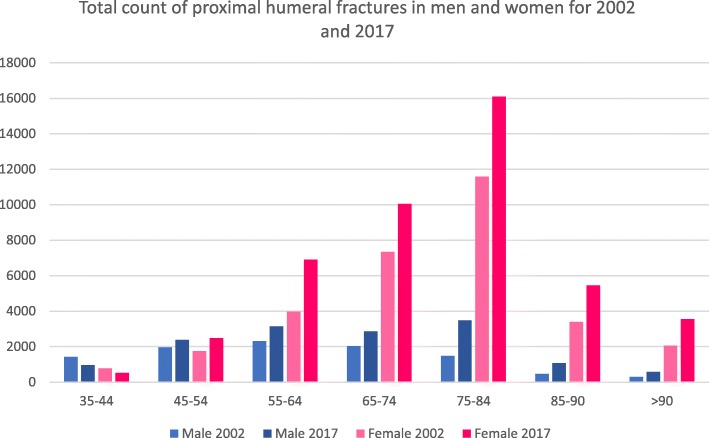
Total count of proximal humeral fractures in men and women for 2002 and 2017

**Fig. 2:**
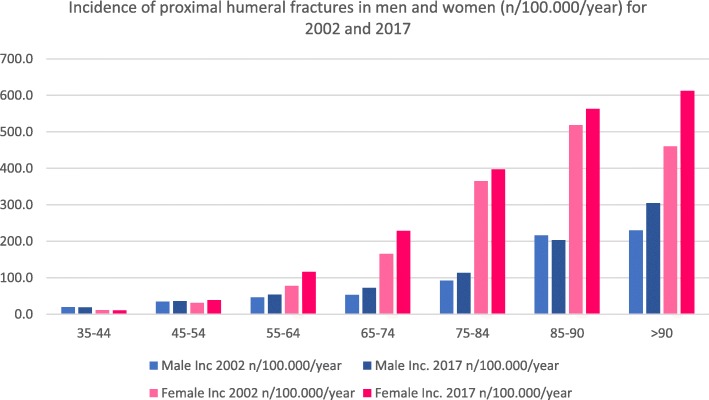
Incidence of proximal humeral fractures in men and women (*n*/100,000/year) for 2002 and 2017

Forty thousand eight hundred thirty-nine fractures were registered (men/women 9967/30,872) in the year 2002. Data showed an increase to 59,545 fractures (men/women 14,484/45,061) in 2017 (46%). Further evaluation showed that the part of female fractures was 75.6% and men 24.4% in 2002, while nearly the same distribution with 75.7% women and 24.3% men occurred in 2017 (Fig. 1). Total counts, incidences and changes between 2017 and 2002 are presented in Table 1. The increase could be stated in all age groups, except the young ones. Especially in the male age group of 75–84 years, data showed a high increase with 137%. Similar could be stated for males in the age group of 85–90 years. Women aged 75 years and older showed a two to three times higher incidence of proximal humerus fractures than men. Figure 2 shows also that the incidence is rising in both genders.

**Table 1:** A total count, differences between 2017 and 2002 in percent and incidences for 2002 and 2017 for proximal humerus fractures for men and women. Total count and incidences for patients > 90 years have been added together

Proximal humerus fracture S42.2	Age (years)	Total count		Ratio (%)	Incidence (*n*/100,000/year)	
		2002	2017	2017:2002	2002	2017
Male	35–44	1416	962	− 32	19.7	19.1
	45–54	1961	2381	21	34.4	36.5
	55–64	2316	3135	35	46.4	53.8
	65–74	2033	2865	41	53.6	72.8
	75–84	1470	3486	137	92.0	113.5
	85–90	471	1072	128	216.8	203.3
	> 90	300	583	94	229.7	304.8
Female	35–44	770	530	− 31	11.3	10.8
	45–54	1749	2480	42	31.2	38.8
	55–64	3973	6910	74	77.8	116.1
	65–74	7333	10,050	37	165.8	229.1
	75–84	11,587	16,095	39	365.2	397.5
	85–90	3399	5453	60	518.2	563.2
	> 90	2061	3543	72	460.1	612.2

### Distal humerus

**Fig. 3:**
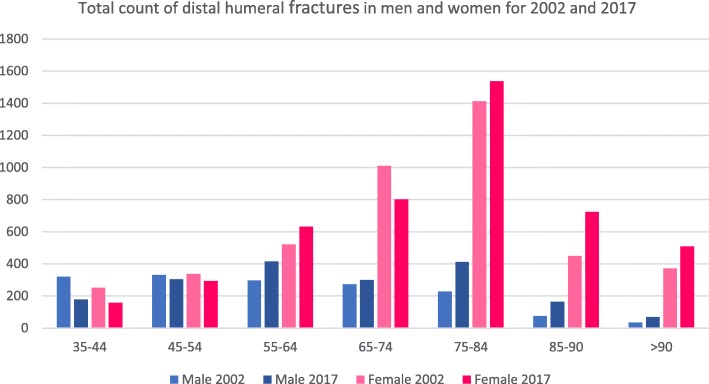
Total count of distal humeral fractures in men and women for 2002 and 2017

**Fig. 4:**
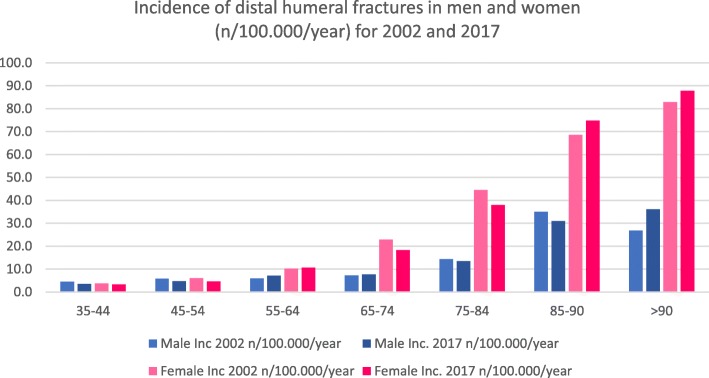
Incidence of distal humeral fractures in men and women (*n*/100,000/year) for 2002 and 2017

Five thousand nine hundred twelve fractures were registered in 2002 located at the distal humerus (men/women 1559/4353). In 2017, a total count of 6493 fractures (men/women 1840/4653) was described with an increase of 9%. The part of female distal humerus fractures in 2002 was 73.6% (men 26.4%). In 2017, the percentage of females with distal humeral fractures decreased to 71.7% (men 28.3%; Table 2) (Figs. 3 and 4). The youngest age groups showed a decrease of fracture incidences and total counts between 2002 and 2017. The three oldest male age groups showed the strongest ratio increase.

**Table 2:** Total count, differences between 2017 and 2002 in percent and incidences for 2002 and 2017 for distal humerus fractures for men and women. Total count and incidences for patients > 90 years have been added together [16]

Distal humerus fracture S42.4	Age (years)	Total count		Ratio (%)	Incidence (*n*/100,000/year)	
		2002	2017	2017:2002	2002	2017
Male	35–44	321	178	− 45	4.5	3.5
	45–54	330	304	− 8	5.8	4.7
	55–64	296	414	40	5.9	7.1
	65–74	273	300	10	7.2	7.6
	75–84	228	412	81	14.3	13.4
	85–90	76	163	114	35.0	30.9
	> 90	35	69	97	26.8	36.1
Female	35–44	251	158	− 37	3.7	3.2
	45–54	338	294	− 13	6.0	4.6
	55–64	521	631	21	10.2	10.6
	65–74	1010	802	− 21	22.8	18.3
	75–84	1412	1536	9	44.5	37.9
	85–90	450	724	61	68.6	74.8
	> 90	371	508	37	82.8	87.8

### Forearm

**Fig. 5:**
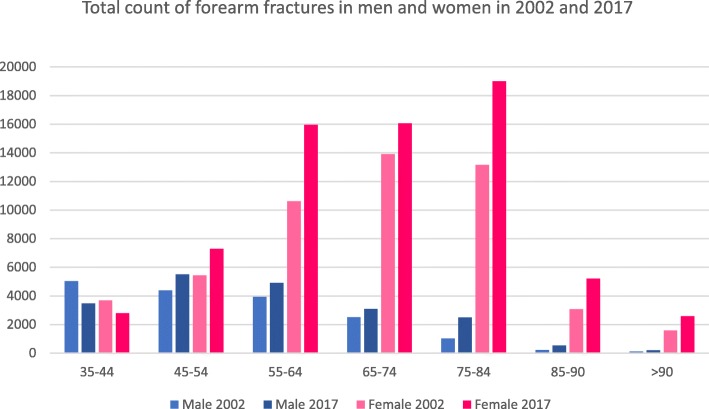
Total count of forearm fractures in men and women for 2002 and 2017

**Fig. 6:**
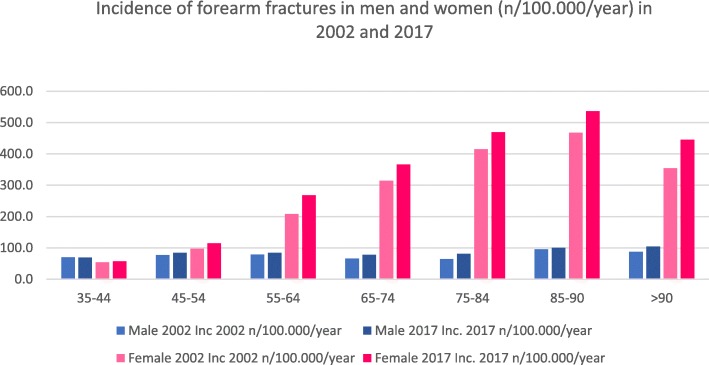
Incidence of forearm fractures in men and women (*n*/100,000/year) for 2002 and 2017

In 2002, a total count of 68,636 fractures were registered (men/women 17,186/women 51,450), whereas 89,040 fractures were registered (men/women 20,185/68,855) in the year 2017 with a total increase of 30%. In 75.0%, female patients suffered from those fractures in 2002 with an increase of 2.3% until 2017 (men 22.7%). Typical geriatric fractures like the distal radial fracture showed an increase in both sexes in every age group. Nearly every fracture occurrence of the forearm (proximal, diaphysis, distal) increased in different age groups in both sexes. Table 3 shows the different age groups and the corresponding fracture distribution. Figures 5 and 6 show total counts and incidences summarised for all forearm fractures.

**Table 3:** Total count, differences between 2017 and 2002 in percent and incidences for 2002 and 2017 for fractures of the forearm for men and women. Absolute numbers and incidences for patients > 90 years have been added together [16]

	Age (years)	Fracture of the	ICD-10-Code	Total count		Ratio (%)	Incidence (*n*/100,000/year)	
				2002	2017	2017:2002	2002	2017
Male	35–44	Proximal ulna	S52.0	776	392	− 49	10.8	7.8
		Proximal radius	S52.1	920	729	− 21	12.8	14.5
		Ulna diaphysis	S52.2	323	190	− 41	4.5	3.8
		Distal radius	S52.5	3005	2159	− 28	41.8	43.0
	45–54	Proximal ulna	S52.0	679	726	7	11.9	11.1
		Proximal radius	S52.1	591	874	48	10.4	13.4
		Ulna diaphysis	S52.2	268	286	7	4.7	4.4
		Distal radius	S52.5	2839	3617	27	49.9	55.5
	55–64	Proximal ulna	S52.0	628	759	21	12.6	13.0
		Proximal radius	S52.1	305	535	75	6.1	9.2
		Ulna diaphysis	S52.2	217	272	25	4.3	4.7
		Distal radius	S52.5	2780	3343	20	55.7	57.4
	65–74	Proximal ulna	S52.0	431	494	15	11.4	12.5
		Proximal radius	S52.1	101	205	103	2.7	5.2
		Ulna diaphysis	S52.2	132	126	− 5	3.5	3.2
		Distal radius	S52.5	1845	2260	22	48.6	57.4
	75–84	Proximal ulna	S52.0	250	477	91	15.7	15.5
		Proximal radius	S52.1	26	107	312	1.6	3.5
		Ulna diaphysis	S52.2	35	107	206	2.2	3.5
		Distal radius	S52.5	714	1798	152	44.7	58.5
	85–90	Proximal ulna	S52.0	62	136	119	28.5	25.8
		Proximal radius	S52.1	5	15	200	2.3	2.8
		Ulna diaphysis	S52.2	8	13	63	3.7	2.5
		Distal radius	S52.5	132	366	177	60.7	69.4
	> 90	Proximal ulna	S52.0	41	65	59	31.4	34.0
		Proximal radius	S52.1	8	5	− 38	6.1	2.6
		Ulna diaphysis	S52.2	3	6	100	2.3	3.1
		Distal radius	S52.5	62	123	98	47.5	64.3
Female	35–44	Proximal ulna	S52.0	446	236	− 47	6.5	4.8
		Proximal radius	S52.1	612	492	− 20	9.0	10.0
		Ulna diaphysis	S52.2	163	86	− 47	2.4	1.7
		Distal radius	S52.5	2459	1983	− 19	36.0	40.2
	45–54	Proximal ulna	S52.0	514	611	19	9.2	9.6
		Proximal radius	S52.1	710	1007	42	12.7	15.7
		Ulna diaphysis	S52.2	167	186	11	3.0	2.9
		Distal radius	S52.5	4037	5491	36	72.0	85.9
	55–64	Proximal ulna	S52.0	979	1076	10	19.2	18.1
		Proximal radius	S52.1	855	1372	60	16.7	23.0
		Ulna diaphysis	S52.2	216	303	40	4.2	5.1
		Distal radius	S52.5	8561	13,195	54	167.6	221.7
	65–74	Proximal ulna	S52.0	1302	1273	− 2	29.4	29.0
		Proximal radius	S52.1	542	838	55	12.3	19.1
		Ulna diaphysis	S52.2	247	287	16	5.6	6.5
		Distal radius	S52.5	11,823	13,656	16	267.2	311.3
	75–84	Proximal ulna	S52.0	1704	1927	13	53.7	47.6
		Proximal radius	S52.1	270	532	97	8.5	13.1
		Ulna diaphysis	S52.2	211	312	48	6.7	7.7
		Distal radius	S52.5	10,977	16,223	48	346.0	400.6
	85–90	Proximal ulna	S52.0	481	717	49	73.3	74.1
		Proximal radius	S52.1	59	75	27	9.0	7.7
		Ulna diaphysis	S52.2	57	81	42	8.7	8.4
		Distal radius	S52.5	2471	4318	75	376.8	446.0
	> 90	Proximal ulna	S52.0	296	471	59	66.1	81.4
		Proximal radius	S52.1	39	39	0	8.7	6.7
		Ulna diaphysis	S52.2	39	56	44	8.7	9.7
		Distal radius	S52.5	1213	2012	66	270.8	347.6

## Discussion

The goal of this study was to analyse trends in fracture development of the upper extremity in people aged > 35 years in Germany. Main findings were an increase of total counts and incidences for proximal and distal humeral fractures especially in older age groups in both sexes. Forearm fractures also showed an increase for both sexes aged over 45 years and the majority of patients suffering from fractures of the upper extremity were female.

There is a lack of epidemiological data for fractures of the upper extremity in the Federal Republic of Germany. There are register studies that investigated fractures of the upper extremity in Sweden and Finland [7, 13, 14]. Sumrein et al. showed, e.g. that mostly females suffer from a proximal humeral fractures with an incidence of 73% in Sweden [14] and Jo et al. reported about 78% female patients of their overall cohort in South Korea [17]. This study shows similar results with 75% females affected by proximal humeral fractures and a gender distribution of men:women of 3:1. Singer et al. and Hagino et al. reported that the incidence of proximal humerus fractures begins to increase after 50 years of age with a female dominance [18, 19]. We could demonstrate similar results with an increasing total count and incidence for women aged 45 years and older. The rising number of fractures associated with higher age is in accordance with the literature as proximal humeral fractures are also related to fragility fractures [2, 7, 20].

Similar results were seen for distal humeral fractures. Most noticeable is the change from 81 to 114% in men aged > 75 years. However, women still show a higher incidence in every age group > 55 years. Five percent of all osteoporotic low-energy fractures in people aged 60 years or older are distal humeral fractures [13, 21]. The incidence of fractures of adult women increases every decade after the age of 20 [22] and the incidences for distal humeral fractures in German female patients also show an increasing incidence in every decade (see Fig. 4). Bergdahl et al. reported about an increase of incidence after the age of 50 years for men and women which can be related to influencing of age-related risk factors like osteoporosis and a high risk of falls [23]. Distal humeral fractures are usually not regarded as fragility fractures [2]. The strongest change of percentage from 2002 to 2017 (especially in the male group) and an increasing incidence associated with higher age of women suggest that this kind of fracture should also be regarded as a fragility fracture. Court-Brown et al. assumed that distal humeral fractures are osteoporosis related [20]; that is why we think that the high incidence of this fracture type in women associated with the high incidence of osteoporosis in older women in general may strongly support this assumption.

Forearm fractures show a higher incidence for nearly all female age groups compared to men. Until now, fragility fractures of the forearm were mainly focused on the distal radius [7, 24]. Almost every fracture of the forearm increased in incidence in both sexes > 65 years of age. Especially, the absolute numbers and incidence for women > 55 years showed a strong increase. Court-Brown et al. also registered an increasing incidence of ulna diaphyseal fractures in females [2]. An explanation could be a higher life expectation associated with better health and a higher activity level in everyday life. This is confirmed on the one hand by the increased total count of fractures as well as the increased incidence (see Table 3). Nevertheless, a more specific view for fractures of the forearm seems to be necessary. Therefore, these fractures should be reclassified [2] particularly under the aspect of trauma mechanism and age.

Our results showed that demographic change [1] is also reflected by fracture development. Total counts and incidences of typical fragility fractures increased as well as the total spectrum of upper extremity fractures. It seems that there are several reasons why women suffer more often than men from the investigated fractures. Firstly, the amount of old people grows (related to higher life expectancy) and especially the number of older women [25]. Secondly, elderly people are more active than in the past [26]. Thirdly, women suffer from osteoporosis up to 5 times more often than men [27] and osteoporosis manifests earlier in women. As a consequence, fractures occur at a much earlier stage of life and predominantly in women. Women live longer with restrictions due to longer life expectancy and risk of recurring falls [28, 29].

However, men showed an increasing number of fractures, too. They also have a better life expectancy and reach higher ages. Furthermore, it should be considered that men also suffer from osteoporosis. The German osteoporosis guideline group recommends basic diagnostics generally for women and men starting at the age of 70 years due to the increased risk of fractures [30].

The present study has got several limitations. The national hospital discharge diagnosis register only captures inpatients. The number of outpatients is not registered which probably increases the absolute numbers of fractures and incidences as well. Additionally, the register does not give any information about fracture classifications and therefore the fracture severity. Mistakes in fracture coding could also be possible. Furthermore, there is no difference between surgically and conservatively treated patients. Another limitation is the lack of recording of osteoporosis diagnoses in relation to the fractures.

This study highlights several problems. Trauma surgeons will have to deal with a growing number of upper extremity fractures in multimorbid as well as active geriatric patients in a probably more complex and demanding general setting as proximal humerus fractures, e.g. show a more complex fracture morphology in elderly patients than in the past years [31]. The authors could demonstrate that this also applies to nearly all fractures of the upper extremity. Health care will therefore be confronted with an increasing fracture rate of geriatric patients and consecutively with a growing financial burden. Another reason is that many patients have multiple side diagnoses. Therefore, interdisciplinary cooperation between trauma surgeons, geriatricians, nursing staff and rehabilitation specialists is necessary [32]. The high number of women suffering from fractures suggests that a sufficient osteoporosis diagnosis and therapy is still not performed although there are several prevention programmes. The German health care planning and hospital-based therapeutic strategies should focus more on the geriatric patient population.

## Conclusion

This is the first study that describes the fracture development of the upper extremity in Germany for patients aged > 35 years in the years 2002 and 2017. The general assumption so far that fragility fractures only describe proximal humeral and distal radial fractures does not seem to be accurate [2]. Especially, fractures of the distal humerus and forearm occur more often in elderly patients than 15 years ago.

## Data Availability

The datasets used and/or analysed during the current study are available from the corresponding author on reasonable request.

## Abbreviations

ICD: International Classification of Diseases
ICD-10-GM: International Classification of Diseases 10th version German Modification
RKI: Robert Koch Institute
